# Pulmonary Effective Arterial Elastance by Echocardiography and Mortality in the Cardiac Intensive Care Unit

**DOI:** 10.1016/j.jacadv.2025.101806

**Published:** 2025-05-28

**Authors:** Mitchell Padkins, Garvan Kane, Jeremy Thaden, Joseph G. Murphy, Michael A. Solomon, Meir Tabi, Christopher Barnett, Jacob C. Jentzer

**Affiliations:** a Department of Cardiovascular Medicine, Mayo Clinic, Rochester, Minnesota, USA; b Critical Care Medicine Department, National Institutes of Health Clinical Center and Cardiovascular Branch, National Heart, Lung, and Blood Institute of the National Institutes of Health, Bethesda, Maryland, USA; c Heart Institute, H’aEmek Medical Center, Faculty of Medicine, Technion-Israel Institute of Technology, Haifa, Israel; d Division of Cardiology, Department of Medicine, University of California-San Francisco, San Francisco, California, USA

**Keywords:** cardiac intensive care unit, pulmonary arterial effective elastance, shock

## Abstract

**Background:**

Elevated right ventricular systolic pressure (RVSP) is associated with higher mortality in cardiac intensive care unit (CICU) patients. Markers of right ventricular-pulmonary artery (PA) coupling may be superior to RVSP.

**Objectives:**

The authors sought to determine whether effective PA elastance (E_PA_, RVSP to stroke volume ratio) and the ratio of pulmonary and systemic elastances (RVSP to systolic blood pressure [SBP] ratio) predicted mortality in a CICU population.

**Methods:**

Mayo Clinic CICU admissions from 2007 to 2018 with available data for E_PA_ or RVSP/SBP were included. The primary outcome was in-hospital mortality, and predictors of in-hospital mortality were analyzed using multivariable logistic regression.

**Results:**

The included 5,004 unique CICU patients had a median age of 72.2 years; 40.9% were females. The 348 (7.7%) patients who died during hospitalization had higher E_PA_ (0.75 vs 0.51) and RVSP/SBP ratio (0.44 vs 0.33). Greater values of E_PA_ (adjusted OR: 1.12 per 0.1 higher, 95% CI: 1.09-1.16) and RVSP/SBP (adjusted OR: 1.18 per 0.1 higher, 95% CI: 1.11-1.25) ratios were incrementally associated with higher severity of illness, more comorbidities, and increased in-hospital mortality. One-year mortality was incrementally higher with increasing values of E_PA_ (adjusted HR: 1.09 per 0.1 higher, 95% CI: 1.08-1.1) and RVSP/SBP ratio (adjusted HR: 1.09 per 0.1 higher, 95% CI: 1.07-1.1). Both E_PA_ and RVSP/SBP ratio had higher discrimination than RVSP alone for predicting in-hospital mortality.

**Conclusions:**

Noninvasive echocardiographic E_PA_ and RVSP/SBP ratio can be used to incrementally prognosticate among CICU patients, and these parameters predict mortality better than RVSP alone.

Among cardiac intensive care unit (CICU) patients, the presence of pulmonary hypertension (PH) and right ventricular (RV) dysfunction are important predictors of higher mortality.^1^, ^2^, ^3^, ^4^ This is in keeping with the importance of RV dysfunction and preload as drivers of secondary end-organ injury in patients with circulatory failure.^5^^,^^6^ A limitation in the use of RVSP to predict mortality in critically ill patients is the observation that RVSP can fall due to RV failure masking the extent of pulmonary vascular disease. When evaluating patients with PH and RV dysfunction, it is crucial to appreciate that pulmonary artery (PA) systolic and diastolic pressures, pulmonary vascular resistance, and transpulmonary gradients are all flow-dependent hemodynamic parameters that can paradoxically decrease in the setting of RV failure. Accordingly, echocardiographic markers of right ventricular-pulmonary artery (RV-PA) coupling may be superior to simply estimating the PA pressure for predicting CICU mortality.^3^

The effective PA elastance (E_PA_), easily calculated by indexing the right ventricular systolic pressure (RVSP) to the stroke volume (SV), is a promising marker of RV-PA coupling and PA stiffness.^7^, ^8^, ^9^, ^10^, ^11^ The E_PA_ reflects both the resistive and pulsatile load from the RV to the PA, encompassing total RV afterload.^11^^,^^12^ Importantly, the E_PA_ may unmask high RV afterload that has resulted in loss of RV-PA coupling consequent on RV dysfunction with a secondary reduction in RVSP resulting in a drop in SV due to poor RV cardiac output and forward flow (Central Illustration). E_PA_ can be derived in different ways, but this simple derivation is consistent with prior studies and has been shown to prognosticate and predict mortality in critically ill populations.^9^^,^^10^

**Central Illustration:**
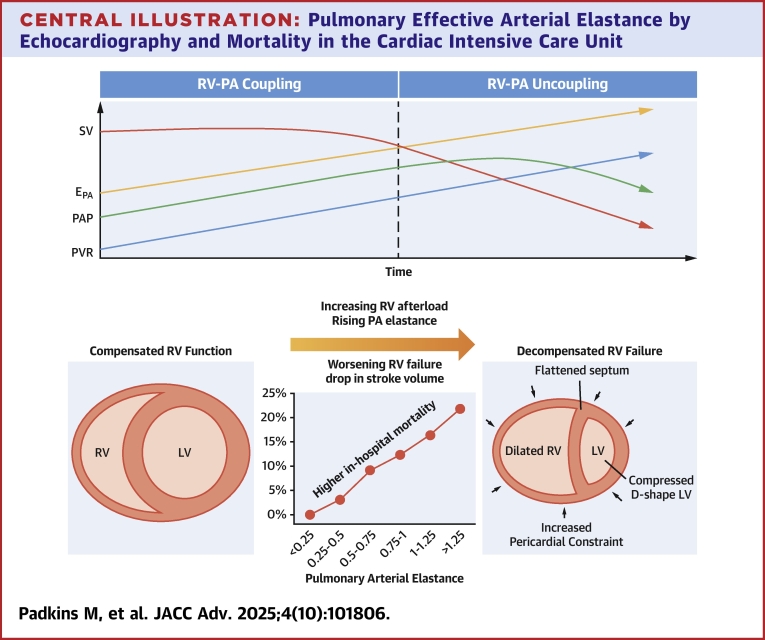
**Pulmonary Effective Arterial Elastance by Echocardiography and Mortality in the Cardiac Intensive Care Unit** The top illustration demonstrates RV-PA coupling on the left with satisfactory RV function. The right portion demonstrates a reduction in SV due to RV-PA uncoupling and RV dysfunction. The bottom illustration demonstrates in-hospital mortality stratified by PA elastance and RVSP/SBP quartiles and the physiologic and hemodynamic changes of the right ventricle with RV-PA uncoupling. EPA = pulmonary effective arterial elastance; LV = left ventricular; PA = pulmonary artery; PAP = pulmonary arterial pressure; PVR = pulmonary vascular resistance; RV = right ventricular; RVSP = right ventricular systolic pressure; SBP = systolic blood pressure; SV = stroke volume.

Systemic arterial effective elastance (E_a_), defined as systolic blood pressure (SBP) divided by SV, predicts outcomes in multiple cardiac pathologies including shock, heart failure (HF), coronary artery disease, and valvular abnormalities.^13^, ^14^, ^15^ The E_a_ is thought to reflect arterial load on the left ventricle and overall left ventricular afterload similarly to E_PA_ for the RV.^14^^,^^16^ The ratio of E_PA_ to E_a_ (ie, the pulmonary to systemic elastance ratio) mathematically simplifies to RVSP/SBP and can provide insights into effective right and left ventricular elastances and relative afterload. Accordingly, we tested the hypothesis that E_PA_ and the ratio of ventricular elastances (RVSP/SBP) estimated noninvasively using echocardiography would predict mortality in an unselected CICU population.

## Methods

This retrospective observational cohort study was approved by the Mayo Clinic Institutional Review Board (IRB # 16-000722), including a waiver of informed consent due to the minimal risk nature of the study under the Declaration of Helsinki. This was a historical cohort analysis using a secondary analysis of a previously constructed institutional database of patients admitted to the CICU at the Mayo Clinic Hospital, St. Mary’s Campus. Patients who did not provide research consent authorization were excluded from the study in accordance with Minnesota State law.

Data were retrospectively analyzed from the index CICU admission of consecutive unique adult patients aged ≥18 years admitted to the CICU at Mayo Clinic Hospital St. Mary’s Campus between January 1, 2007, and April 30, 2018, who had an echocardiogram within 1 day of CICU admission. Patients who did not have available hemodynamic data for either E_PA_ or RVSP/SBP were excluded. From the echocardiographic data, the RVSP was estimated using the tricuspid valve regurgitant velocity, if an adequate signal was present, plus the right atrial pressure (RAP). Inferior vena cava size and collapsibility were used to estimate the RAP, with invasively measured RAP substituted if available.^2^^,^^3^^,^^17^, ^18^, ^19^ Left ventricular SV was calculated using the left ventricular outflow tract diameter and pulsed-wave Doppler as described by the American Society of Echocardiography guidelines.^20^ The E_PA_ was calculated as RVSP/SV, and the RVSP/SBP ratio was calculated based on vital signs from the time of transthoracic echocardiogram (TTE). Semiquantitative RV function was graded based on the overall assessment of the interpreting cardiologist incorporating all available data.^4^

We electronically extracted demographic, vital signs, laboratory, clinical and outcome data, procedures, and therapies performed during the index CICU and hospital stay.^21^, ^22^, ^23^, ^24^, ^25^, ^26^, ^27^, ^28^ The admission vital signs, clinical measurements, and laboratory values were defined as the first value recorded after or closest to the index CICU admission. Admission diagnoses were defined as all International Classification of Diseases-9/10 diagnostic codes documented within 1 day before or after the index CICU admission; these were not mutually exclusive.^25^, ^26^, ^27^, ^28^, ^29^

The Acute Physiology and Chronic Health Evaluation (APACHE)-III score, APACHE-IV predicted hospital mortality, and Sequential Organ Failure Assessment scores were automatically calculated using data from the first 24 hours of CICU admission using previously validated electronic algorithms.^22^^,^^24^^,^^26^^,^^30^^,^^31^ The Mayo Clinic CICU Admission Risk Score (M-CARS) was calculated on admission as previously described.^28^ Individual comorbidities used to calculate the Charlson Comorbidity Index were extracted from the medical record using a previously validated electronic algorithm. The Braden Skin Score was used as a surrogate marker of frailty.^24^^,^^28^^,^^32^

All-cause CICU, in-hospital mortality, and 1-year mortality were determined using an electronic review of medical records for notification of patient death. Categorical variables are reported as numbers (percentages), and the Pearson chi-square test was used to compare groups. Continuous variables are reported as median (IQR), and the Kruskal-Wallis rank-sum test was used to compare groups. Discrimination was assessed using the area under the receiver-operator characteristic curve (AUC, C-statistic) values. OR and 95% CI values for in-hospital mortality were calculated using logistic regression, before and after multivariable adjustment. One-year survival was evaluated using Kaplan-Meier curves, with groups compared using the log-rank test. HR and 95% CI values for 1-year mortality were calculated using Cox proportional-hazards regression, before and after multivariable adjustment. Proportionality of hazards was tested using the Schoenfeld test and Schoenfeld residual plots. Multivariable models were adjusted for the following established predictors of mortality in this CICU population as done in previous analyses from our group, selected a priori: age, sex, Charlson Comorbidity Index, APACHE-III score, positive-pressure ventilation, number of vasoactive drugs, use of coronary angiography/PCI, and admission diagnoses of acute coronary syndrome, HF, shock, cardiac arrest, respiratory failure, and sepsis.^2^^,^^25^^,^^33^ All *P* values were two-tailed. Statistical analyses were performed using BlueSky version 7.4 (BlueSky LLC).

## Results

### Study population

Out of a database of 12,428 unique CICU patients, 7,444 had a TTE within 1 day of CICU admission. Among these patients, 5,004 had available data for E_PA_ (n = 4,508) or RVSP/SBP ratio (n = 4,939) and were included in the final study population. The final study population had a median age of 72.2 (60.3, 80.5) years, and 40.9% were females. Prior lung disease was documented in 18.9%, and positive-pressure ventilation was used in 28.8%. Admission diagnoses (not mutually exclusive) included ACS (53.2%), HF (53.1%), respiratory failure (26.6%), and cardiogenic shock (14.2%). Median left ventricular ejection fraction was 50% (35%, 61%). Moderate or severe RV dysfunction was present in 31% of patients with available data (n = 3,218). Median RVSP was 39 mm Hg, and 61.1% met criteria for PH (estimated RVSP ≥35 mm Hg).^34^ The median E_PA_ was 0.52 (0.37-0.75), and median RVSP/SBP ratio was 0.34 (0.26-0.45).

When patients were divided by quartiles of E_PA_ (Table 1), substantial differences in baseline characteristics were noted. Patients with higher E_PA_ were more often females, had higher rates of comorbidities (including HF or lung disease), had more critical care admission diagnoses (eg, shock or respiratory failure), and greater overall illness severity and utilization of critical care therapies (eg, positive-pressure ventilation or vasoactive drugs). Patients with higher E_PA_ had more extensive echocardiographic abnormalities, including worse biventricular function, lower SV, and higher filling pressures. When patients were divided by quartiles of RVSP/SBP ratio (Table 2), similar differences in baseline characteristics were observed. Patients with higher RVSP/SBP ratios were similar to patients with higher E_PA_, resulting in parallel trends in clinical and echocardiographic findings across quartiles.

**Table 1:** Baseline Characteristics of Cohort Stratified by E_PA_ Quartile and Overall

	PA Elastance Quartiles					
	1 (n = 1,128)	2 (n = 1,127)	3 (n = 1,126)	4 (n = 1,127)	Total (n = 4,508)	*P* Value
	0.134-0.368	0.369-0.522	0.523-0.753	0.754-4.714	0.134-4.714	
Demographics						
Age, y	65.4 (55.3, 75.6)	71.7 (61.8, 80.5)	75.1 (64.5, 83.3)	72.6 (62.1, 82.9)	71.3 (60.5, 80.7)	<0.001
Female	259 (23%)	448 (39.8%)	520 (46.2%)	591 (52.4%)	1,818 (40.3%)	<0.001
White race	1,060 (94%)	1,066 (94.6%)	1,038 (92.2%)	1,014 (90%)	4,178 (92.7%)	<0.001
Charlson Comorbidity Index	1 (0, 2)	1 (0, 3)	2 (1, 4)	2 (1, 4)	2 (0, 3)	<0.001
Hospital length of stay (d)	3.0 (2.1, 5.0)	3.9 (2.7, 6.8)	5.6 (3.3, 9.3)	7.1 (4.2, 12.1)	4.6 (2.8, 8.5)	<0.001
ICU length of stay (d)	1.47 (0.9, 2.2)	1.77 (1.0, 2.8)	2 (1.1, 3.3)	2.4 (1.2, 4.2)	1.9 (1.0, 3.0)	<0.001
Hospital mortality	19 (1.7%)	49 (4.3%)	106 (9.4%)	174 (15.4%)	348 (7.7%)	<0.001
CICU mortality	9 (0.8%)	31 (2.8%)	57 (5.1%)	117 (10.4%)	214 (4.7%)	<0.001
Comorbidities						
Myocardial infarction	170 (15.1%)	208 (18.5%)	231 (20.5%)	207 (18.4%)	816 (18.1%)	<0.001
Heart failure	80 (7.1%)	150 (13.3%)	240 (21.3%)	348 (30.9%)	818 (18.2%)	<0.001
Cerebrovascular accident	88 (7.8%)	125 (11.1%)	159 (14.1%)	162 (14.4%)	534 (11.9%)	<0.001
Moderate/severe kidney disease	118 (10.5%)	205 (18.2%)	264 (23.5%)	284 (25.2%)	871 (19.3%)	<0.001
Dialysis	17 (1.5%)	43 (3.8%)	68 (6%)	72 (6.4%)	200 (4.4%)	<0.001
Diabetes mellitus	260 (23.1%)	293 (26%)	368 (32.7%)	356 (31.6%)	1,277 (28.4%)	<0.001
Cancer	202 (18%)	249 (22.1%)	283 (25.2%)	247 (21.9%)	981 (21.8%)	<0.001
Lung disease	135 (12%)	189 (16.8%)	252 (22.4%)	255 (22.6%)	831 (18.5%)	<0.001
Admission diagnoses						
Cardiac arrest	94 (8.4%)	129 (11.6%)	146 (13%)	167 (14.9%)	536 (12%)	<0.001
Shock	63 (5.7%)	109 (9.8%)	211 (18.8%)	331 (29.4%)	714 (16%)	<0.001
Sepsis	20 (1.8%)	56 (5%)	105 (9.4%)	120 (10.7%)	301 (6.7%)	<0.001
Respiratory failure	95 (8.5%)	202 (18.2%)	395 (35.2%)	445 (39.6%)	1,137 (25.4%)	<0.001
CHF	249 (22.4%)	460 (41.4%)	735 (65.5%)	922 (82%)	2,366 (52.9%)	<0.001
Acute coronary syndrome	681 (61.2%)	659 (59.4%)	600 (53.5%)	486 (43.2%)	2,426 (54.3%)	<0.001
Cardiogenic shock	51 (4.6%)	81 (7.3%)	163 (14.5%)	294 (26.2%)	589 (13.2%)	<0.001
Severity of illness						
Apache 3 score	48 (36-60)	57 (44-71)	64 (52-79)	68 (55-82)	59 (46-74)	<0.001
Apache 4 predicted mortality	0.0 (0.0-0.1)	0.1 (0.0-0.2)	0.1 (0.1-0.3)	0.2 (0.1-0.3)	0.1 (0.0-0.2)	<0.001
Braden Skin Score	19 (17-21)	18 (16-20)	17 (15-20)	17 (15-19)	18 (16-20)	<0.001
M-CARS	1 (0-2)	1 (0-3)	2 (1-4)	3 (2-5)	2 (0-3)	<0.001
Shock index	0.55 (0.5-0.7)	0.6 (0.5-0.7)	0.7 (0.5-0.8)	0.8 (0.6-0.9)	0.6 (0.5-0.8)	<0.001
Procedures and therapeutics						
Any ventilator	145 (12.9%)	242 (21.5%)	384 (34.1%)	464 (41.2%)	1,235 (27.4%)	<0.001
Invasive ventilator	64 (5.7%)	128 (11.4%)	233 (20.7%)	255 (22.6%)	680 (15.1%)	<0.001
Noninvasive ventilator	95 (8.4%)	152 (13.5%)	224 (19.9%)	280 (24.8%)	751 (16.7%)	<0.001
Vasopressor medications	95 (8.4%)	155 (13.8%)	275 (24.4%)	368 (32.7%)	893 (19.8%)	<0.001
Inotropic medications	10 (0.9%)	35 (3.1%)	81 (7.2%)	208 (18.5%)	334 (7.4%)	<0.001
Dialysis	12 (1.1%)	26 (2.3%)	57 (5.1%)	89 (7.9%)	184 (4.1%)	<0.001
Intra-aortic balloon pump	59 (5.2%)	67 (5.9%)	113 (10%)	163 (14.5%)	402 (8.9%)	<0.001
Pulmonary artery catheter	36 (3.2%)	42 (3.7%)	98 (8.7%)	201 (17.8%)	377 (8.4%)	<0.001
Coronary angiogram	795 (70.5%)	707 (62.7%)	667 (59.2%)	631 (56%)	2,800 (62.1%)	<0.001
Percutaneous coronary intervention	587 (52%)	523 (46.4%)	408 (36.2%)	264 (23.4%)	1,782 (39.5%)	<0.001
Echocardiographic parameters						
LV ejection fraction (%)	57 (48-63)	53 (40-61)	45 (32-57)	35 (22-55)	50 (35-60.1)	<0.001
LV end-diastolic dimension (mm)	51 (47-55)	50 (46-56)	51 (46-57)	52 (45-59)	51 (46-56)	0.08
LV end-systolic dimension (mm)	34 (30-38)	35 (30-41)	37 (31-45)	40 (31-51)	35 (30-43)	<0.001
Interventricular septal thickness (mm)	11 (10-12)	11 (10-12)	11 (10-12)	11 (9-12)	11 (10-12)	0.03
Posterior wall thickness (mm)	10 (9-11)	10 (9-11)	10 (9-12)	10 (9-11)	10 (9-11)	0.002
Relative wall thickness (%)	0.42 (0.38-0.47)	0.42 (0.37-0.47)	0.42 (0.36-0.49)	0.41 (0.34-0.48)	0.42 (0.37-0.48)	0.011
LV outflow tract velocity PWD velocity (m/s)	1.1 (1-1.2)	1 (0.9-1.1)	1 (0.9-1.1)	0.9 (0.7-1)	1 (0.9-1.1)	<0.001
LVOT velocity time integral PWD (cm)	22 (20-25)	20.3 (18-23.3)	19 (16-22)	14.9 (12-18)	19.3 (16-23)	<0.001
LV stroke volume (mL)	96 (83-108)	81 (71-94)	69 (60-82)	53 (42-64)	76 (60-91)	<0.001
LV stroke work index (mm Hg/mL/m^2^)	45.2 (37.9-54.1)	38.3 (32.1-46.2)	31.4 (25.2-38.9)	24.1 (18-30.1)	35.9 (27.1-45.3)	<0.001
LV cardiac output (L/min)	6.15 (5.3-7.1)	5.67 (4.8-6.7)	5.2 (4.4-6.3)	4.4 (3.7-5.3)	5.4 (4.4-6.5)	<0.001
LV cardiac index (L/min/m^2^)	3 (2.6-3.5)	2.9 (2.5-3.4)	2.8 (2.4-3.3)	2.4 (2-2.8)	2.8 (2.4-3.3)	<0.001
Cardiac power output (W)	1.2 (1-1.4)	1 (0.8-1.3)	0.9 (0.7-1.1)	0.8 (0.6-1)	1 (0.8-1.2)	<0.001
Cardiac power index (W/m^2^)	0.6 (0.5-0.7)	0.5 (0.4-0.6)	0.5 (0.4-0.6)	0.4 (0.3-0.5)	0.5 (0.4-0.6)	<0.001
Medial e' (m/s)	6 (5-8)	6 (5-7)	5 (4-6)	5 (4-6)	6 (4-7)	<0.001
Lateral e' (m/s)	8 (7-10)	7 (6-9)	7 (5-9)	7 (5-9)	7 (6-9)	<0.001
Mitral valve E wave PWD (m/s)	0.7 (0.6-0.9)	0.8 (0.6-1.0)	0.9 (0.7-1.1)	0.9 (0.7-1.2)	0.8 (0.6-1.0)	<0.001
Mitral valve E/A ratio	1 (0.8-1.3)	1 (0.8-1.3)	1.1 (0.8-1.6)	1.3 (0.9-2)	1.1 (0.8-1.5)	<0.001
Mitral valve deceleration time (m)	201 (176-237)	189 (163-227)	170.5 (146.8-203)	150 (128-181)	181 (152-218)	<0.001
Medial E/e'	10 (8.3-14)	13.3 (10-18)	16.7 (12-23.3)	18 (13.3-26.7)	14 (10-20)	<0.001
Lateral E/e'	8.6 (6.7-11.2)	10 (8-14)	12.5 (9.1-18)	13.3 (10-20)	10 (7.8-15)	<0.001
TV regurgitation velocity continuous wave Doppler (CWD) (m/s)	2.4 (2.2-2.5)	2.6 (2.4-2.9)	2.9 (2.6-3.2)	3.2 (2.8-3.5)	2.7 (2.4-3.1)	<0.001
RAP (estimated)	5 (5-10)	5 (5-10)	10 (5-14)	14 (10-20)	10 (5-14)	<0.001
RVSP (estimated) (mm Hg)	28 (24-32)	36 (31-41)	44 (37-51)	54 (46-64)	39 (31-50)	<0.001
Right ventricular stroke work index (mm Hg/mL/m^2^)	1,035 (816-1,325)	1,160 (897-1,496)	1,224 (868-1,710)	1,132.5 (745-1,662)	1,125 (828-1,548)	<0.001
Tricuspid annular systolic velocity (m/s)	12 (10-14)	12 (10-14)	11 (9-13)	10 (7-12)	11 (9-13)	<0.001
Pulmonary artery elastance	0.3 (0.27-0.34)	0.4 (0.4-0.5)	0.6 (0.6-0.7)	1.0 (0.8-1.2)	0.5 (0.4-0.8)	<0.001
Systolic blood pressure (mm Hg)	117 (106-132)	118 (105-134)	114 (101-129)	110 (98-127)	115 (102-130)	<0.001
Diastolic blood pressure (mm Hg)	66 (57-75)	63 (55-73)	61 (53-70.5)	64 (55-74)	64 (55-73)	<0.001
Heart rate (beats/min)	66 (58-74)	71 (61-81)	75 (65-88)	85 (73-100)	73 (63-86)	<0.001
Pulse pressure (mm Hg)	51 (41-63)	53 (42-68)	51 (40-65)	45 (34-59)	50 (39-64)	<0.001
MAP (mm Hg)	83.3 (74.7-92.7)	82 (73.3-91.7)	79.3 (70.7-89.2)	79.3 (70.7-90.6)	81.3 (73.3-91)	<0.001
RAP/pulmonary capillary wedge pressure ratio	0.4 (0.3-0.4)	0.4 (0.3-0.6)	0.4 (0.3-0.6)	0.5 (0.3-0.8)	0.4 (0.3-0.6)	<0.001
RAP/MAP ratio	0.1 (0.1-0.1)	0.1 (0.1-0.1)	0.1 (0.1-0.2)	0.2 (0.1-0.2)	0.1 (0.1–0.2)	<0.001
RVSP/SBP ratio	0.2 (0.2-0.3)	0.3 (0.3-0.4)	0.4 (0.3-0.4)	0.5 (0.4-0.6)	0.3 (0.3–0.4)	<0.001

Values are n (%) or median (IQR).

CHF = congestive heart failure; CICU = cardiac intensive care unit; E_PA_ = effective pulmonary arterial elastance; LV = left ventricular; M-CARS = Mayo Clinic CICU Admission Risk Score; MAP = mean arterial pressure; PA = pulmonary artery; PWD = pulsed-wave Doppler; RAP = right atrial pressure; RV = right ventricular; RVSP = right ventricular systolic pressure; SBP = systolic blood pressure; TV = tricuspid valve.

**Table 2:** Baseline Characteristics of Cohort Stratified by RVSP/SBP and Overall

	RVSP/SBP Ratio					
	1 (n = 1,235)	2 (n = 1,235)	3 (n = 1,235)	4 (n = 1,234)	Total (n = 4,939)	*P* Value
	0.115-0.263	0.264-0.342	0.342-0.448	0.449-4.000	0.115-4.000	
Demographics						
Age	66.8 (56.4-77.7)	71.2 (60.6-79.9)	74.1 (62.4-82.4)	72.9 (63.4-82.0)	71.2 (60.4-80.6)	<0.001
Female	441 (35.7%)	487 (39.4%)	551 (44.6%)	540 (43.8%)	2,019 (40.9%)	<0.001
White race	1,148 (93%)	1,145 (92.7%)	1,156 (93.6%)	1,128 (91.4%)	4,577 (92.7%)	0.2
Charlson Comorbidity Index	1 (0-3)	1 (0-3)	2 (0-4)	2 (1-4)	2 (0-4)	<0.001
Hospital length of stay (d)	3.18 (2.13-5.61)	4.03 (2.64-7.27)	5.58 (3.18-9.43)	6.96 (3.98-12.41)	4.73 (2.82-8.7)	<0.001
ICU length of stay (d)	1.48 (0.92-2.37)	1.75 (1.01-2.78)	2.06 (1.15-3.45)	2.24 (1.13-4.05)	1.87 (1.03-3.05)	<0.001
CICU mortality	11 (0.9%)	50 (4%)	67 (5.4%)	132 (10.7%)	260 (5.3%)	<0.001
Comorbidities						
MI	190 (15.4%)	235 (19%)	229 (18.6%)	245 (19.9%)	899 (18.2%)	0.025
HF	91 (7.4%)	167 (13.5%)	263 (21.3%)	380 (30.8%)	901 (18.3%)	<0.001
CVA	109 (8.8%)	140 (11.3%)	173 (14%)	172 (13.9%)	594 (12%)	<0.001
Moderate/severe kidney disease	163 (13.2%)	188 (15.2%)	272 (22%)	330 (26.7%)	953 (19.3%)	<0.001
Dialysis	27 (2.2%)	39 (3.2%)	70 (5.7%)	96 (7.8%)	232 (4.7%)	<0.001
Diabetes mellitus	278 (22.6%)	331 (26.8%)	355 (28.8%)	427 (34.6%)	1,391 (28.2%)	<0.001
Cancer	241 (19.6%)	234 (19%)	325 (26.3%)	283 (22.9%)	1,083 (21.9%)	<0.001
Lung disease	147 (11.9%)	209 (16.9%)	279 (22.6%)	297 (24.1%)	932 (18.9%)	<0.001
Admission diagnoses						
Cardiac arrest	111 (9.1%)	151 (12.3%)	173 (14.1%)	178 (14.5%)	613 (12.5%)	<0.001
Shock	82 (6.7%)	166 (13.6%)	255 (20.8%)	340 (27.7%)	843 (17.2%)	<0.001
Sepsis	28 (2.3%)	70 (5.7%)	115 (9.4%)	141 (11.5%)	354 (7.2%)	<0.001
Respiratory failure	137 (11.2%)	290 (23.7%)	388 (31.6%)	492 (40.1%)	1,307 (26.7%)	<0.001
HF	315 (25.8%)	555 (45.4%)	787 (64.1%)	941 (76.6%)	2,598 (53%)	<0.001
Acute coronary syndrome	730 (59.9%)	734 (60%)	636 (51.8%)	510 (41.5%)	2,610 (53.3%)	<0.001
Cardiogenic shock	61 (5%)	138 (11.3%)	214 (17.4%)	288 (23.5%)	701 (14.3%)	<0.001
Severity of illness						
Apache 3 score	49 (36-61)	57 (44-71)	64 (52-79)	69 (55-84)	59 (46-74)	<0.001
Apache 4 predicted mortality	0.05 (0.02-0.11)	0.09 (0.04-0.19)	0.14 (0.07-0.28)	0.17 (0.08-0.35)	0.11 (0.05-0.23)	<0.001
Braden Skin Score	19 (17-21)	18 (16-20)	17 (15-20)	17 (14-19)	18 (15-20)	<0.001
M-CARS	1 (0-2)	1 (0-3)	2 (1-4)	3 (2-5)	2 (0-3)	<0.001
Shock index	0.54 (0.46-0.64)	0.61 (0.51-0.75)	0.68 (0.55-0.82)	0.79 (0.65-0.96)	0.64 (0.52-0.8)	<0.001
Procedures and therapeutics						
Any ventilator	172 (13.9%)	304 (24.6%)	425 (34.4%)	523 (42.4%)	1,424 (28.8%)	<0.001
Invasive ventilator	91 (7.4%)	170 (13.8%)	244 (19.8%)	298 (24.1%)	803 (16.3%)	<0.001
Noninvasive ventilator	101 (8.2%)	180 (14.6%)	251 (20.3%)	319 (25.9%)	851 (17.2%)	<0.001
Vasopressor medications	120 (9.7%)	206 (16.7%)	281 (22.8%)	433 (35.1%)	1,040 (21.1%)	<0.001
Inotropic medications	18 (1.5%)	51 (4.1%)	105 (8.5%)	207 (16.8%)	381 (7.7%)	<0.001
Dialysis	18 (1.5%)	28 (2.3%)	61 (4.9%)	108 (8.8%)	215 (4.4%)	<0.001
Intra-aortic balloon pump	83 (6.7%)	109 (8.8%)	125 (10.1%)	142 (11.5%)	459 (9.3%)	<0.001
Pulmonary artery catheter	45 (3.6%)	62 (5%)	123 (10%)	207 (16.8%)	437 (8.8%)	<0.001
Coronary angiogram	838 (67.9%)	794 (64.3%)	729 (59%)	653 (52.9%)	3,014 (61%)	<0.001
Percutaneous coronary intervention	615 (49.8%)	562 (45.5%)	432 (35%)	302 (24.5%)	1,911 (38.7%)	<0.001
Echocardiographic parameters						
LV ejection fraction (%)	55 (45-63)	50 (36-60)	45 (31-59)	44 (27-60)	50 (35-61)	<0.001
LVEDD (mm)	50 (46-54)	50 (46-55)	52 (46-57)	52 (46-59)	51 (46-56)	<0.001
LVESD (mm)	34 (30-38)	35 (30-41)	37 (31-46)	38 (30-49)	35 (30-43)	<0.001
Interventricular septal thickness (mm)	11 (10-12)	11 (10-12)	11 (10-12)	11 (10-12)	11 (10-12)	0.57
Posterior wall thickness (mm)	10 (9-11)	10 (9-11)	10 (9-12)	10 (9-12)	10 (9-11)	0.14
Relative wall thickness (%)	0.42 (0.38-0.47)	0.42 (0.38-0.48)	0.41 (0.36-0.48)	0.41 (0.35-0.48)	0.42 (0.37-0.48)	<0.001
LVOT velocity PWD (m/s)	1 (0.9-1.1)	1 (0.9-1.1)	1 (0.9-1.1)	1 (0.8-1.1)	1 (0.9-1.1)	<0.001
LVOT VTI PWD (cm)	21 (18-23)	20 (16.9-23)	19 (15.1-22)	17.3 (14-21)	19.2 (16-23)	<0.001
LV stroke volume (mL)	83 (71-98)	79 (63-95)	72 (57.25-87)	66 (51-83)	76 (60-91)	<0.001
LV stroke work index (mL/m^2^)	43.86 (36.1-53.36)	37.64 (30.33-45.32)	32.56 (25.18-40.92)	26.37 (19.61-33.78)	35.88 (27.13-45.26)	<0.001
LV cardiac output (L/min)	5.6 (4.81-6.55)	5.44 (4.46-6.53)	5.25 (4.32-6.39)	5.13 (4.15-6.36)	5.37 (4.43-6.47)	<0.001
LV cardiac index (L/min/m^2^)	2.87 (2.51-3.26)	2.79 (2.41-3.27)	2.72 (2.33-3.33)	2.69 (2.18-3.28)	2.76 (2.38-3.29)	<0.001
Cardiac power output (watts)	1.11 (0.91-1.34)	0.99 (0.80-1.23)	0.93 (0.73-1.16)	0.84 (0.66-1.05)	0.97 (0.76-1.21)	<0.001
Cardiac power index (watts/m^2^)	0.56 (0.47-0.67)	0.51 (0.42-0.61)	0.48 (0.39-0.60)	0.43 (0.35-0.55)	0.5 (0.4-0.62)	<0.001
Medial e' (m/sec)	6 (5-8)	6 (4-7)	5 (4-7)	5 (4-6)	6 (4-7)	<0.001
Lateral e' (m/sec)	8 (6-10)	8 (6-10)	7 (5-9)	7 (5-9)	7 (6-9)	<0.001
Mitral valve E wave PWD (m/s)	0.7 (0.6-0.9)	0.8 (0.6-1)	0.9 (0.7-1.1)	1 (0.8-1.2)	0.8 (0.6-1)	<0.001
Mitral valve E/A ratio	1 (0.71-1.25)	1 (0.75-1.4)	1.2 (0.8-1.75)	1.25 (0.89-2)	1.08 (0.75-1.5)	<0.001
Mitral valve deceleration time (m/s)	197 (172-239)	190 (161-224)	171.5 (145-207.75)	159 (134-190.5)	181 (152-219)	<0.001
Medial E/e'	11.25 (8.57-15)	13.33 (10-18)	16 (11.43-22.5)	18.33 (13.33-26.67)	14 (10-20)	<0.001
Lateral E/e'	8.75 (7-11.67)	10 (7.78-14)	12 (8.57-16.67)	13.33 (10-20)	10 (7.78-15)	<0.001
TV regurgitation velocity CWD (m/s)	2.3 (2.14-2.5)	2.6 (2.4-2.8)	2.9 (2.6-3.1)	3.3 (3-3.6)	2.7 (2.4-3.1)	<0.001
RAP (estimated)	5 (5-5)	5 (5-10)	10 (10-14)	14 (10-20)	10 (5-14)	<0.001
RVSP (estimated) (mm Hg)	28 (24-30)	35 (32-39)	44 (39-50)	56 (49-66)	39 (31-50)	<0.001
RVSWI (mm Hg/mL/m^2^)	900 (720-1,131)	1,100 (840-1,395)	1,240 (899-1,656)	1,485 (1,058-2,024)	1,122 (828-1,540)	<0.001
TASV (m/sec)	12 (10-14)	12 (9.25-14)	11 (8-13)	10 (8-13)	11 (9-13)	<0.001
PA elastance	0.33 (0.28-0.41)	0.46 (0.37-0.58)	0.62 (0.49-0.79)	0.87 (0.68-1.16)	0.52 (0.37-0.75)	<0.001
Systolic blood pressure (mm Hg)	126 (114-140)	118 (106-131.5)	113 (100-127)	102 (92-114)	115 (102-130)	<0.001
Diastolic blood pressure (mm Hg)	70 (61-79)	64 (56-73)	62 (54-72)	58 (50-67)	63 (55-73)	<0.001
Heart rate (beats/min)	69 (60-78.75)	72 (62-85)	76 (64-89)	80 (69-96)	74 (63-88)	<0.001
Pulse pressure (mm Hg)	55 (44-70)	51 (41-65)	50 (38-64)	43 (33-55)	50 (39-63)	<0.001
MAP (mm Hg)	88.67 (80.33-97.83)	82.67 (74.33-91.33)	79.33 (70.67-88.67)	73.33 (65.33-81.67)	81 (72-90.83)	<0.001
RAP/PCWP ratio	0.35 (0.27-0.43)	0.35 (0.25-0.57)	0.47 (0.29-0.7)	0.51 (0.34-0.77)	0.4 (0.28-0.61)	<0.001
RAP/MAP ratio	0.06 (0.05-0.07)	0.08 (0.06-0.13)	0.14 (0.1-0.19)	0.19 (0.14-0.25)	0.11 (0.06-0.17)	<0.001
RVSP/SBP ratio	0.22 (0.2-0.24)	0.3 (0.28-0.32)	0.39 (0.36-0.42)	0.53 (0.49-0.62)	0.34 (0.26-0.45)	<0.001

Values are median (IQR) or n (%).

CICU = cardiac intensive care unit; CVA = cerebrovascular accident; CWD = continuous wave Doppler; HF = heart failure; ICU = intensive care unit; LV = left ventricular; LVEDD = LV end-diastolic dimension; LVESD = LV end-systolic dimension; LVOT = left ventricular outflow tract; MAP = mean arterial pressure; M-CARS = Mayo Clinic CICU Admission Risk Score; MI = myocardial infarction; PA = pulmonary artery; PCWP = pulmonary capillary wedge pressure; PWD = pulsed-wave Doppler; RAP = right atrial pressure; RVSP = right ventricular systolic pressure; RVSWI = right ventricular stroke work index; SBP = systolic blood pressure; TASV = tricuspid annular systolic velocity; TV = tricuspid valve; VTI = velocity time integral.

### In-hospital mortality

**Figure 1:**
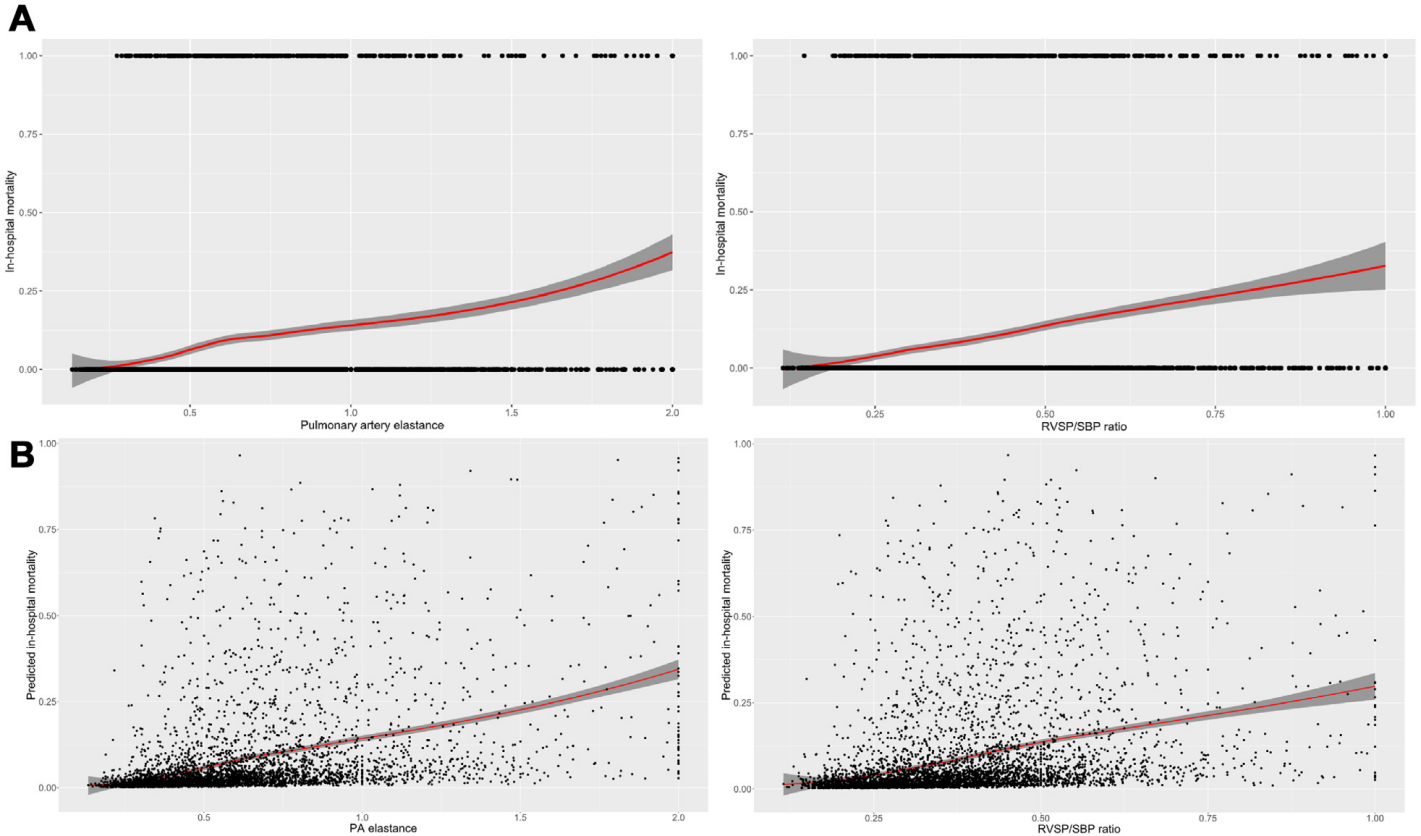
Association Between E_PA_ and RVSP/SBP Ratio With In-Hospital Mortality Association between (A) E_PA_ or (B) RVSP/SBP ratio with in-hospital mortality. Locally estimated scatterplot smoother (LOESS) curves demonstrating the association between (A) E_PA_ or (B) RVSP/SBP ratio with in-hospital mortality. E_PA_ = effective pulmonary arterial elastance; PA = pulmonary artery; RVSP = right ventricular systolic pressure; SBP = systolic blood pressure.

**Figure 2:**
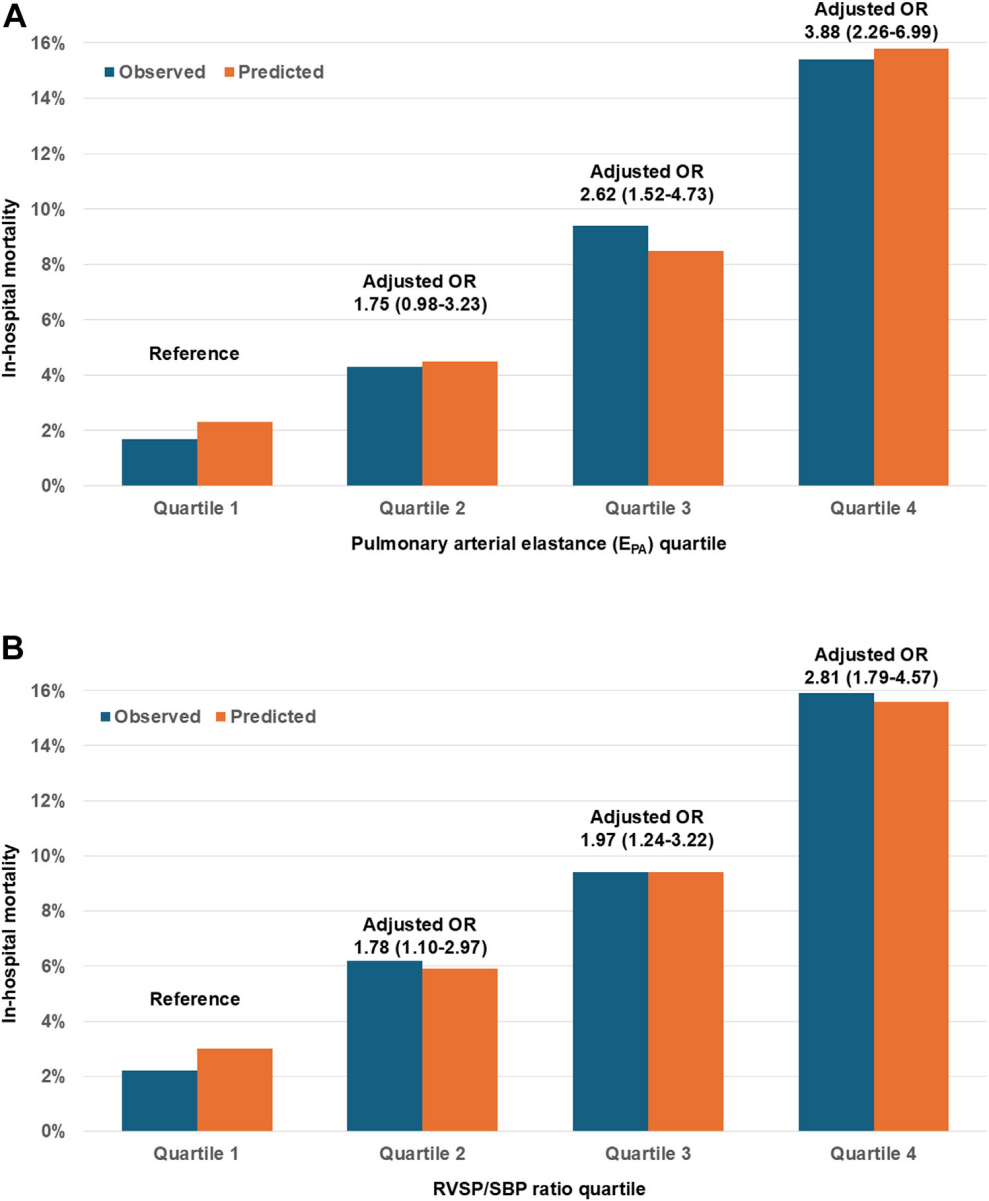
In-Hospital Mortality According to Quartile of E_PA_ or RVSP/SBP Ratio In-hospital mortality according to quartile of (A) E_PA_ or (B) RVSP/SBP ratio. Unadjusted OR and 95% CI values are reported for each higher quartile vs quartile 1 as the reference group. E_PA_ = effective pulmonary arterial elastance; RVSP = right ventricular systolic pressure; SBP = systolic blood pressure.

**Figure 3:**
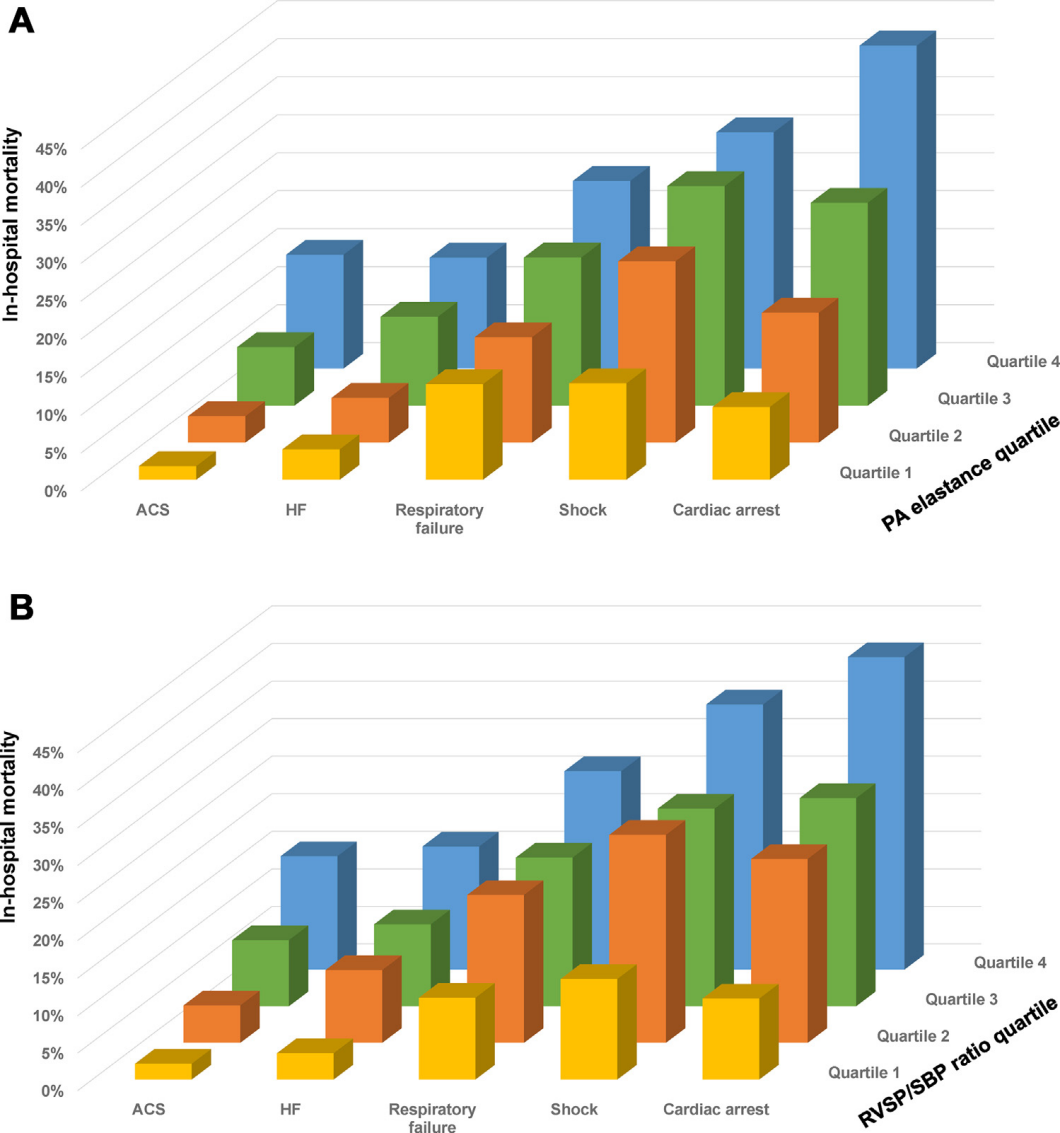
In-Hospital Mortality According to E_PA_ or RVSP/SBP Quartiles Stratified by Admission Diagnoses In-hospital mortality according to (A) E_PA_ or (B) RVSP/SBP quartiles stratified by admission diagnoses. ACS = acute coronary syndrome; E_PA_ = effective pulmonary arterial elastance; HF = heart failure; PA = pulmonary artery; RVSP = right ventricular systolic pressure; SBP = systolic blood pressure.

**Figure 4:**
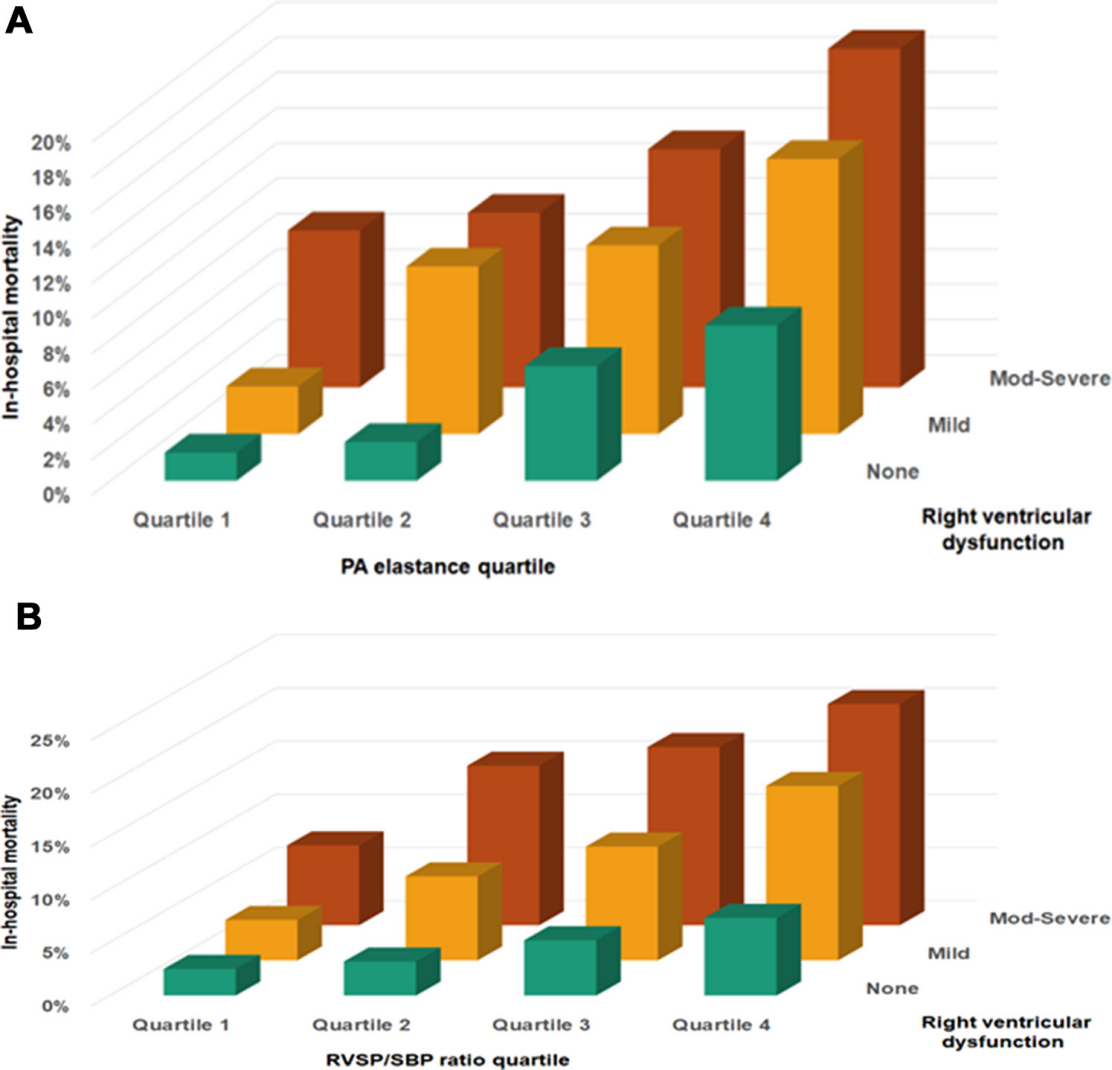
In-Hospital Mortality According to RV Dysfunction Stratified by E_PA_ Quartiles and RVSP/SBP Quartiles In-hospital mortality according to RV dysfunction stratified by (A) E_PA_ quartiles and (B) RVSP/SBP quartiles. E_PA_ = effective pulmonary arterial elastance; PA = pulmonary artery; RV = right ventricular; RVSP = right ventricular systolic pressure; SBP = systolic blood pressure.

A total of 348 (7.7%) patients died during hospitalization, including 214 (4.7%) who died during the CICU stay. Patients who died during hospitalization had higher median values of E_PA_ (0.75 [0.56-1.07] vs 0.51 [0.36-0.73], *P* < 0.001) and RVSP/SBP ratio (0.44 [0.34-0.56] vs 0.33 [0.26-0.44], *P* < 0.001). Linear, incremental associations were observed between greater values of E_PA_ (unadjusted OR: 1.17 per 0.1 higher, 95% CI: 1.14-1.19) (Figure 1A) and RVSP/SBP ratio (unadjusted OR: 1.36 per 0.1 higher, 95% CI: 1.29-1.44) (Figure 1B) and higher crude and adjusted in-hospital mortality (Figure 1). RVSP itself had an incremental association with in-hospital mortality that was not as steep (unadjusted OR: 1.32 per 10 mm Hg higher, 95% CI: 1.25-1.40, *P* < 0.001) (Supplemental Figure 1). As shown in Figures 2A and 2B, crude and adjusted in-hospital mortality was higher in each increasing quartile of either E_PA_ (unadjusted OR per quartile: 2.08, 95% CI: 1.86-2.35) or RVSP/SBP ratio (unadjusted OR per quartile: 1.88, 95% CI: 1.70-2.09). This association remained consistent across admission diagnosis groups (Figures 3A and 3B) and other important subgroups stratified by age, sex, comorbidities, and severity of illness (Supplemental Figure 2). The discrimination C-statistic for in-hospital mortality was higher for E_PA_ than RVSP/SBP ratio (0.72 [0.69-0.75] vs 0.69 [0.66-0.72], *P* = 0.02); both C-statistic values were higher than that for RVSP (0.65 [0.62-0.68], both *P* < 0.001) (Supplemental Figure 3a) but not different from tricuspid annular systolic velocity (TASV)/RVSP ratio (0.72 [0.69-0.75], both *P* > 0.2; Supplemental Figure 3b). In-hospital mortality increased incrementally with each higher quartile of RVSP and each lower quartile of either SV or SBP (Supplemental Figures 3a and 3b). After multivariable adjustment (Table 3), both E_PA_ and RVSP/SBP ratio remained associated with in-hospital mortality when treated as continuous variables or comparing quartiles. These associations persisted after the addition of left ventricular ejection fraction, medial mitral E/e’ ratio, RV S’, or semiquantitative RV dysfunction in the multivariable models (Figures 4A and 4B, Supplemental Figure 4a and 4b).

**Table 3:** Logistic Regression Models for Prediction of In-Hospital Mortality, Before and After Multivariable Adjustment

	Unadjusted Analyses			Adjusted Analyses		
	OR	95% CI	*P* Value	OR	95% CI	*P* Value
PA elastance						
Continuous (per 0.1)	1.166	1.140-1.193	<0.001	1.123	1.090-1.158	<0.001
Each quartile	2.084	1.856-2.349	<0.001	1.531	1.323-1.778	<0.001
Quartile 1	Referent	---	---	Referent	---	---
Quartile 2	2.653	1.579-4.644	<0.001	1.746	0.978-3.233	0.07
Quartile 3	6.066	3.786-10.259	<0.001	2.620	1.518-4.730	<0.001
Quartile 4	10.657	6.762-17.806	<0.001	3.884	2.264-6.985	<0.001
RVSP/SBP ratio						
Continuous (per 0.1)	1.364	1.291-1.442	<0.001	1.175	1.106-1.250	<0.001
Each quartile	1.879	1.697-2.087	<0.001	1.344	1.186-1.527	<0.001
Quartile 1	Referent	---	---	Referent	---	---
Quartile 2	2.934	1.902-4.662	<0.001	1.784	1.098-2.971	0.02
Quartile 3	4.638	3.076-7.244	<0.001	1.969	1.237-3.225	0.005
Quartile 4	8.448	5.704-13.014	<0.001	2.814	1.789-4.568	<0.001

All multivariable models were adjusted for age, sex, CCI, APACHE-III score, positive-pressure ventilation, number of vasoactive drugs, use of coronary angiography/PCI, and admission diagnoses of ACS, heart failure, shock, cardiac arrest, respiratory failure, and sepsis.

ACS = acute coronary syndrome; APACHE = Acute Physiology and Chronic Health Evaluation; CCI = Charlson Comorbidity Index; PA = pulmonary artery; PCI = percutaneous coronary intervention; RVSP = right ventricular systolic pressure; SBP = systolic blood pressure.

#### One-year mortality

**Figure 5:**
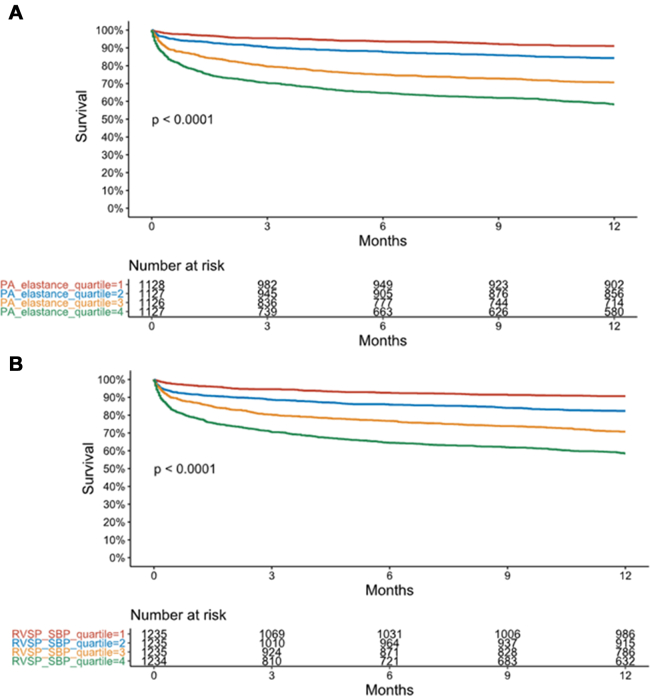
Kaplan-Meier Curves Demonstrating One-Year Survival of Quartiles of E_PA_ or RVSP/SBP Ratio Kaplan-Meier curves demonstrating 1-year survival of quartiles of (A) E_PA_ or (B) RVSP/SBP ratio. E_PA_ = effective pulmonary arterial elastance; PA = pulmonary artery; RVSP = right ventricular systolic pressure; SBP = systolic blood pressure.

A total of 1,157 (23.1%) patients died during 1 year of follow-up, including in-hospital deaths. Among hospital survivors (n = 4,656), postdischarge mortality occurred in 17.4%, and 481 patients were alive at last follow-up but had follow-up <1 year. On Kaplan-Meier analysis, survival was progressively lower with increasing quartile of either E_PA_ (Figure 5A) or RVSP/SBP ratio (Figure 5B). On Cox proportional-hazards analysis, E_PA_ and RVSP/SBP ratio remained associated with 1-year mortality when treated as continuous variables or when comparing quartiles, both before and after multivariable adjustment (Table 4). Discrimination AUC/C-statistic values for 1-year mortality were 0.69 (0.66-0.71) for E_PA_ and 0.68 (0.66-0.70) for RVSP/SBP ratio. Global Schoenfeld tests had low *P* values suggesting violation of the proportional-hazards assumption for the Cox models, but this was minimal for E_PA_ (*P* = 0.04) and RVSP/SBP (*P* = 0.22) with flat Schoenfeld residual plots.

**Table 4:** Cox Proportional-Hazards Models for Prediction of 1-Year Mortality, Before and After Multivariable Adjustment

	Unadjusted Analyses			Adjusted Analyses		
	HR	95% CI	*P* Value	HR	95% CI	*P* Value
PA elastance						
Continuous (per 0.1)	1.113	1.102-1.124	<0.001	1.090	1.075-1.104	<0.001
Each quartile	1.798	1.691-1.912	<0.001	1.424	1.327-1.527	<0.001
Quartile 1	Referent	---	---	Referent	---	---
Quartile 2	1.839	1.427-2.370	<0.001	1.225	0.947-1.586	0.12
Quartile 3	3.788	3.006-4.774	<0.001	1.807	1.415-2.308	<0.001
Quartile 4	5.935	4.746-7.421	<0.001	2.667	2.090-3.404	<0.001
RVSP/SBP ratio						
Continuous (per 0.1)	1.117	1.103-1.130	<0.001	1.090	1.070-1.109	<0.001
Each quartile	1.727	1.631-1.829	<0.001	1.321	1.241-1.406	<0.001
Quartile 1	Referent	---	---	Referent	---	---
Quartile 2	2.008	1.590-2.537	<0.001	1.509	1.193-1.910	<0.001
Quartile 3	3.546	2.854-4.405	<0.001	1.828	1.463-2.286	0.005
Quartile 4	5.563	4.512-6.859	<0.001	2.449	1.966-3.051	<0.001

All multivariable models were adjusted for age, sex, CCI, APACHE-III score, positive-pressure ventilation, number of vasoactive drugs, use of coronary angiography/PCI, and admission diagnoses of ACS, heart failure, shock, cardiac arrest, respiratory failure, and sepsis.

ACS = acute coronary syndrome; APACHE = Acute Physiology and Chronic Health Evaluation; CCI = Charlson Comorbidity Index; PA = pulmonary artery; PCI = percutaneous coronary intervention; RVSP = right ventricular systolic pressure; SBP = systolic blood pressure.

## Discussion

Our study supports the hypothesis that E_PA_ and RVSP/SBP ratio are strongly predictive of mortality in CICU patients and superior to RVSP, a proven predictor of CICU survival. This analysis of over 5,000 unique CICU patients demonstrates that both higher E_PA_ and RVSP/SBP ratio are strongly and incrementally associated with in-hospital mortality across multiple admission diagnoses and provide superior discrimination when compared with the RVSP alone. Our study suggests that whenever the RVSP is calculated by TTE in CICU patients, providers should also calculate the E_PA_ and RVSP/SBP ratio, if possible, to facilitate risk stratification. Both of these easily calculated markers of RV-PA coupling provide incremental risk stratification when added to standard echocardiographic assessment of RV function. By providing insights into the effects of PH on the overall circulation and potentially unmasking concealed PH and low-output RV failure, these easily obtained hemodynamic parameters can refine the prognostication of CICU patients across the spectrum of acute cardiovascular illness. Our results further support the importance of RV-PA coupling as a prognostic determinant in CICU patients and highlight the limitations of a prognostic approach relying solely on the RVSP. While the discrimination AUC C-statistics for these novel TTE parameters may not be high enough to function as standalone prognostication tools, they add meaningfully to risk prediction.

Prior analyses have demonstrated the prognostic significance of E_PA_ in many different clinical circumstances. In patients with PH, the E_PA_ has been found to predict mortality more so than pulmonary vascular resistance or transpulmonary gradient, which are both flow-dependent.^10^ In patients with advanced HF and valvular abnormalities, elevated E_PA_ was associated with greater mortality and has been found to be a good surrogate of RV afterload.^35^^,^^36^ Recent studies have demonstrated that E_PA_ correlated well with RV-PA coupling as demonstrated by 3D echocardiography and cardiac magnetic resonance imaging when compared to the gold standard of cardiac catheterization.^37^ The majority of studies have demonstrated that the increased mortality in precapillary and postcapillary PH is largely due to increased RV load as evidenced by the noninvasive calculation of E_PA_.^10^ Our study is congruent with these prior studies that demonstrate the usefulness of E_PA_, and our findings extend this to prognosticate in the modern CICU population. Our study is both large and unique in that no prior published studies examined the E_PA_ or RVSP/SBP ratio in CICU patients. This study has wide applicability to current CICU and intensive care unit practice demonstrating that E_PA_ is a useful surrogate of RV afterload and RV-PA coupling. Accordingly, we are the first to report the strong prognostic importance of the RVSP/SBP ratio, which approached that of the E_PA_ itself and outperformed the RVSP alone.

Other measures of RV-PA coupling have been proposed, which may be complementary to the E_PA_ and RVSP/SBP ratio, which is more closely tied to RV afterload itself. The most common measures of RV-PA coupling are generated by indexing the RVSP (measured invasively or estimated using TTE) to a longitudinal measure of RV function, either the TASV by tissue Doppler imaging (RV S’/TASV), tricuspid annular plane systolic excursion, or RV longitudinal systolic strain. In a prior analysis from this CICU population, we observed that the RV S’/RVSP ratio was strongly and inversely associated with in-hospital mortality.^3^ In this cohort, the TASV/RVSP ratio expectedly had strong inverse correlations (Pearson r values <−0.6) with both E_PA_ and RVSP/SBP ratio, and the discrimination C-statistics for in-hospital mortality (0.72 [0.69-0.75] for TASV/RVSP ratio) did not differ significantly.

During the progression of PH, as pulmonary vascular resistance rises, the RV can initially compensate, and the RVSP increases progressively as SV is preserved. However, as the RV afterload further increases, loss of RV-PA coupling occurs, and forward flow is compromised with a lower SV and paradoxically stable or decreasing PA pressures; this explains the rationale for the use of E_PA_ as a marker of RV afterload in place of RVSP (Central Illustration). In some clinical settings, stable or decreasing PA pressures may give a false sense of security when instead this may be an ominous sign signifying a failing RV as reflected in the decrease in SV unmasked by a higher E_PA_. Thus, by incorporating systemic hemodynamics, the E_PA_ and RVSP/SBP ratio provide important insights into RV-PA coupling and the effects of high RV afterload on forward flow. The ratio of RVSP/SBP can be thought of as the ratio of the pulmonary (E_PA_) and systemic (E_a_) arterial elastances, reflecting the relative balance in afterload between the ventricles. As calculated in this analysis, E_PA_ and RVSP/SBP ratio are closely linked mathematically, allowing the RVSP/SBP ratio to be substituted when the SV is not available to calculate the more physiologically relevant E_PA_. Other echocardiographic measures of RV-PA coupling, shown to be prognostic in PH cohorts, such as PA capacitance, RV stroke work index, or PA pulsatility index, may also provide value in CICU patients but are more complex as they require an estimation of PA diastolic pressure and hence may be less feasible to calculate noninvasively.^38^^,^^39^ Further studies are needed to identify the effects of therapies commonly used for RV failure (eg, inotropes, vasodilators, and vasopressors) on E_PA_ (as opposed to simply PA pressures) and how changes in E_PA_ over time affect clinical outcomes.

### Study Limitations

This single-center retrospective cohort study has inherent limitations that prevent drawing causal inferences. Selection bias is an important potential cause of confounding, as we excluded patients with missing TTE variables of interest but could not determine why either a TTE was not done or why these measurements were not recorded. The CICU population at Mayo Clinic may differ from other populations in terms of baseline demographics, case mix, and resource utilization. A key challenge in any retrospective cohort study examining outcomes in critically ill patients is the likelihood of residual confounding by unmeasured variables. This is emphasized by the substantial differences in measured characteristics across quartiles of E_PA_ or RVSP/SBP ratio, including age, comorbidities, critical care admission diagnoses, and severity of illness, which all increased across quartiles. Any patient factor that increases the RVSP or decreases the SV (for EPA) or SBP (for RVSP/SBP ratio) will result in an increase in these variables, presumably reflecting worse right heart and systemic hemodynamics. The different sex distribution across EPA quartiles may reflect lower SV in females, as this was not indexed to body size. While we adjusted for a robust array of covariates that encompass the most important aspects of patient condition and collectively had excellent discrimination for mortality, outcomes in this population are affected by numerous other factors we could not account for analytically, such as patient goals of care. Despite this, we found that both E_PA_ and RVSP/SBP ratios remained strongly and incrementally associated with mortality (dose-response effect) when stratified by important baseline characteristics and after adjustment for more than a dozen important variables. To estimate the degree of unmeasured residual confounding that would be required to mitigate our observed associations, we calculated E-value metrics using an online calculator.^40^ For our main logistic regression models predicting in-hospital mortality treating the exposures as continuous variables, the E-values for E_PA_ and RVSP/SBP ratio were 5.8 and 9.5, respectively. This implies that unmeasured residual confounders would need to be very strongly associated (ie, OR > 5-9) with both our exposure and outcome to explain the observed effects. Accordingly, while we suspect that residual confounding may exist and could mitigate the strength of our observed associations, this is unlikely to completely explain our findings.

The calculation of the E_PA_ has nuance and has undergone different derivations, and indeed the optimal approach to calculating E_PA_ for an individual may depend on the shape of the RV pressure-volume loop, which can change as PH and RV failure progress.^9^^,^^11^ However, it has been shown that our simple derivation is relatively accurate and can predict mortality in critically ill populations.^7^^,^^9^ Further, using the systolic PA pressure in the calculation is much easier to obtain and more thoroughly validated using TTE than other derivations using variables such as the mean PA pressure. In addition, our analyses focused only on initial echocardiographic data, and it is possible that changes in these variables over time could provide incremental prognostic value. Further studies would be necessary to determine how changing RV-PA hemodynamics over time would affect a patient’s CICU trajectory and mortality. Data regarding medication administration around the time of TTE (eg, inotropes, pulmonary vasodilators, etc) were unavailable and may have influenced the observed findings. We could not determine which patients were mechanically ventilated at the time of the TTE, which could have affected the measured TTE variables. Our 1-year mortality data should be interpreted with caution due to a potential failure to capture patients dying outside our health system, with potential violation of the proportional-hazards assumption in our Cox models suggesting that our HR estimates may be imprecise. Finally, this cohort primarily included patients with underlying left heart disease and/or lung disease, and our findings therefore apply most directly to those with World Health Organization (WHO) group 2 and/or WHO group 3 PH.^2^^,^^3^^,^^18^

## Conclusions

Our study adds to the growing body of literature demonstrating that a higher E_PA_ predicts a higher risk of mortality across the spectrum of cardiac critical illness. Further, we introduce the novel ratio of pulmonary to systemic arterial elastances (RVSP/SBP) and find that this predicts mortality similarly to E_PA_. E_PA_ and RVSP/SBP were found to predict mortality when stratified by admission diagnoses and biventricular function, and both provide discrimination that exceeds RVSP alone. Thus, these easily obtainable echocardiographic parameters can be used to prognosticate in a CICU cohort. Further research is necessary to determine if changes in these variables over time could better predict mortality and whether specific therapies can influence this association with the goal of improving patient outcomes.

Perspectives **COMPETENCY IN MEDICAL KNOWLEDGE:** During the progression of RV failure, a drop in SV can reduce the measured PA pressures, leading to false reassurance. This can be unmasked by indexing the PA pressure to the SV to calculate the E_PA_.

**COMPETENCY IN PATIENT CARE:** The E_PA_ and the related RVSP to systemic systolic arterial pressure ratio are more strongly associated with mortality in CICU patients than the estimated PA pressure alone.

## Funding support and author disclosures

Dr Solomon has received research support from the 10.13039/100000098National Institutes of Health Clinical Center Intramural research funds. All other authors have reported that they have no relationships relevant to the contents of this paper to disclose.
