# Supplementary material for: Pulmonary Effective Arterial Elastance by Echocardiography and Mortality in the Cardiac Intensive Care Unit
Source: JACC Adv. 2025 May 28;4(10):101806. doi: 10.1016/j.jacadv.2025.101806 (PMC12541210; doi:10.1016/j.jacadv.2025.101806)

**Supplemental Figure 1:** Locally estimated scatterplot smoother (LOESS) curve demonstrating the association between RVSP and in-hospital mortality.


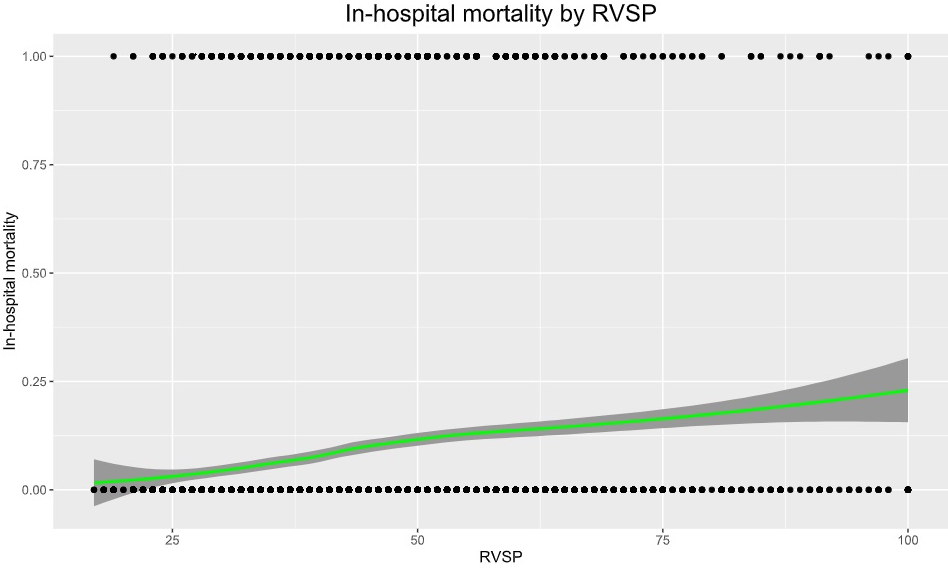


**Supplemental Figure 2:** In-hospital mortality according to quartiles of EPA (a) and RVSP/SBP ratio (b) in subgroups of interest.


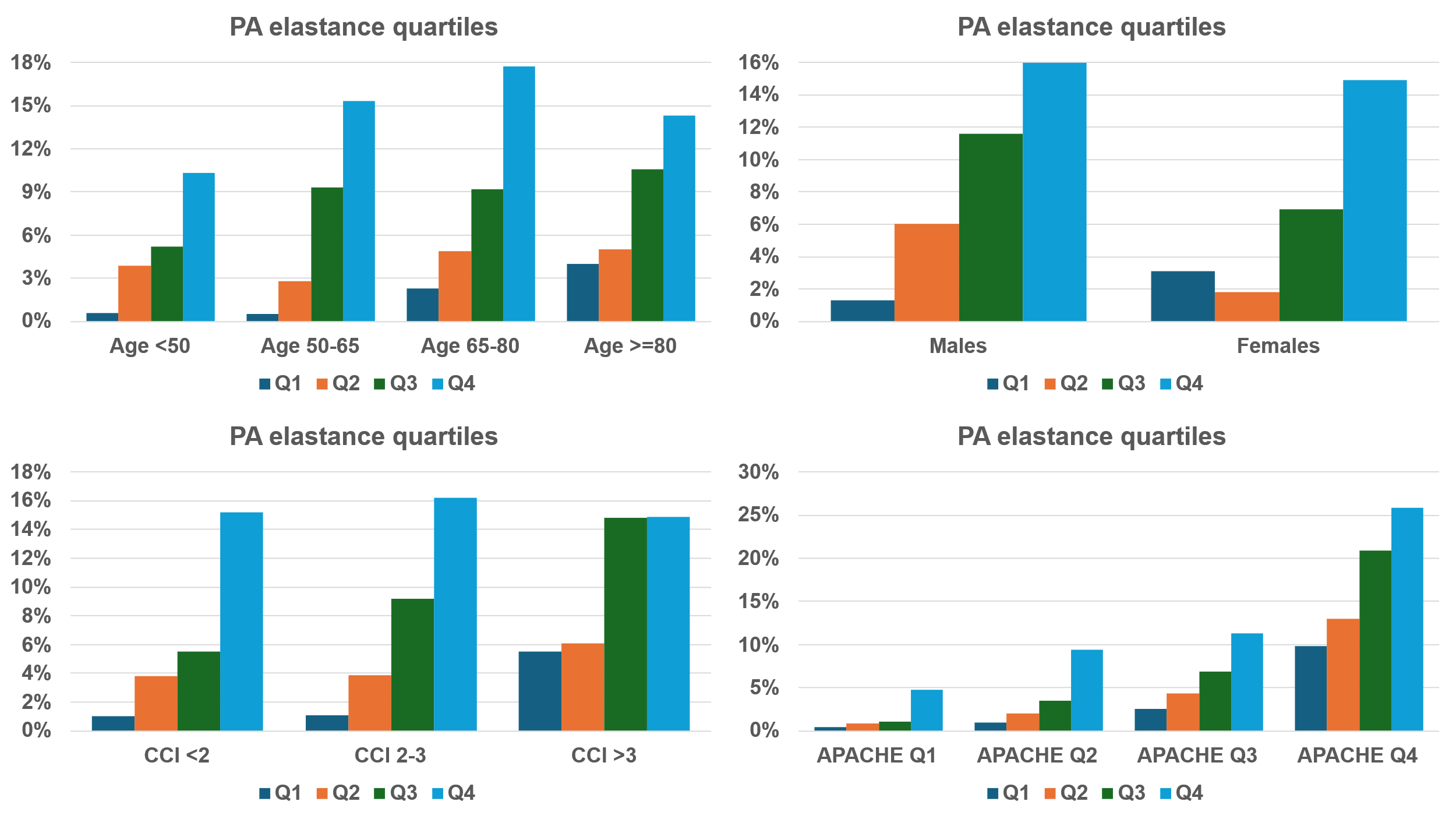


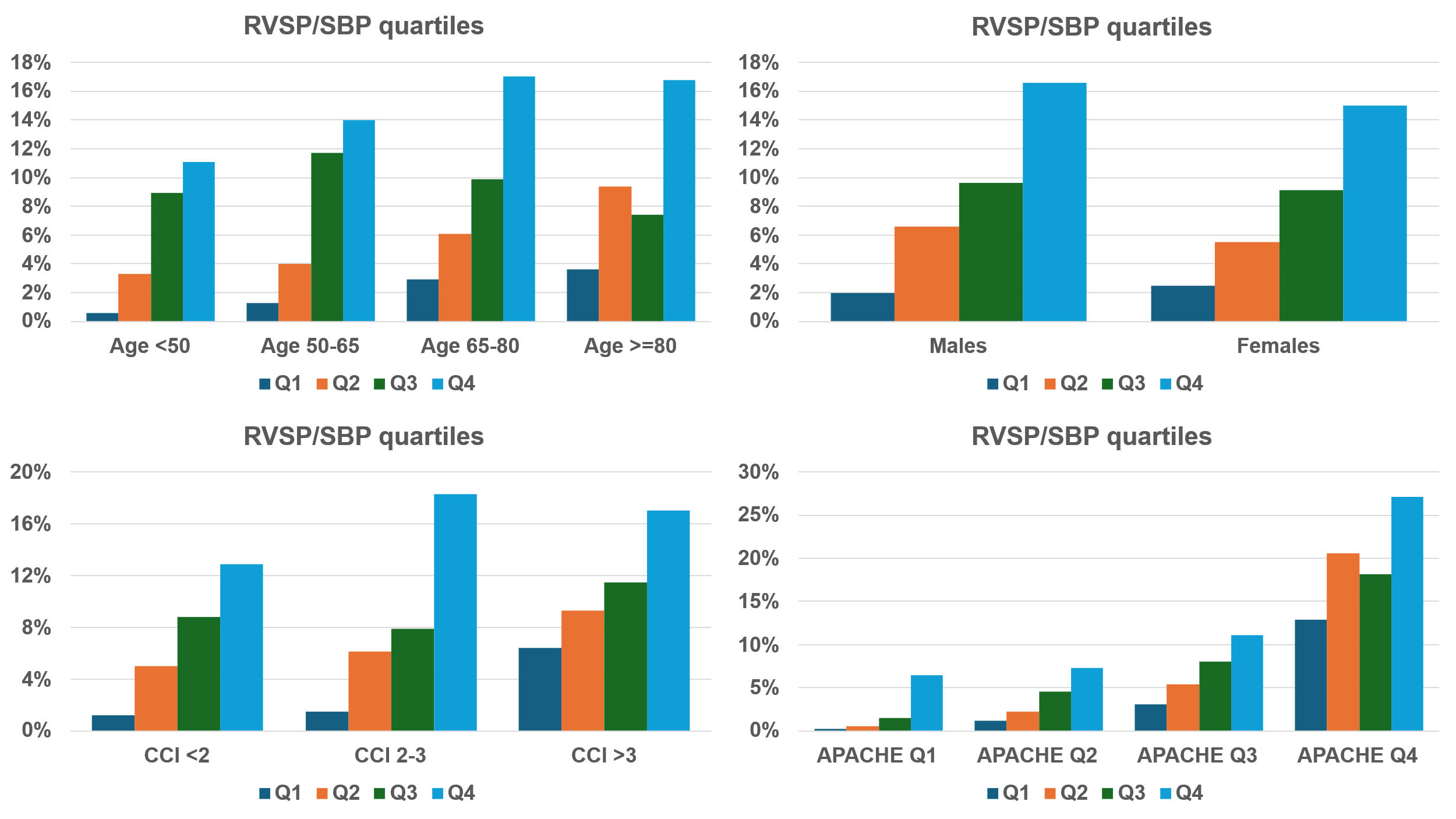


**Supplemental Figure 3:** Receiver operating curve demonstrating the comparison for RVSP, E_PA_, and RVSP/SBP (a) and TASV, E_PA_, and RVSP/SBP (b) for in-hospital mortality.


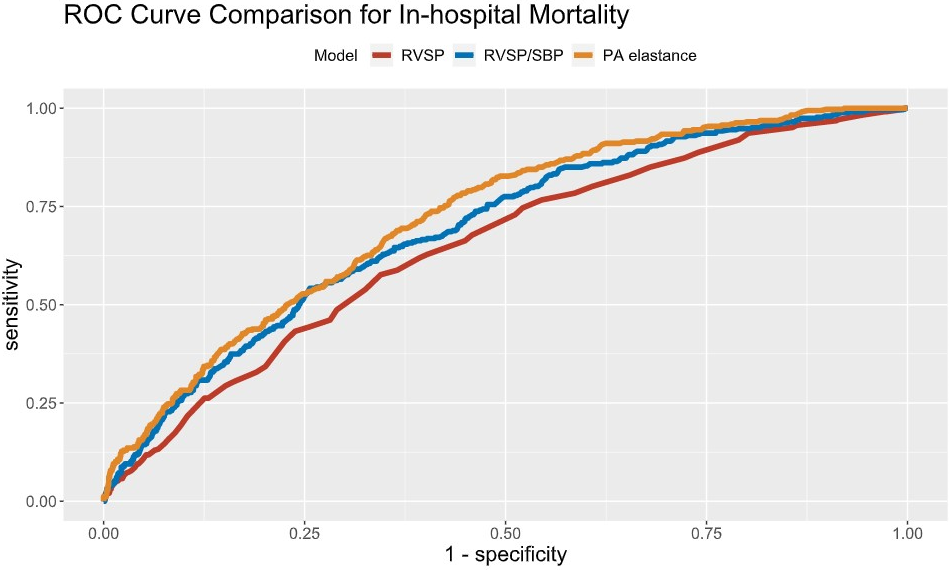


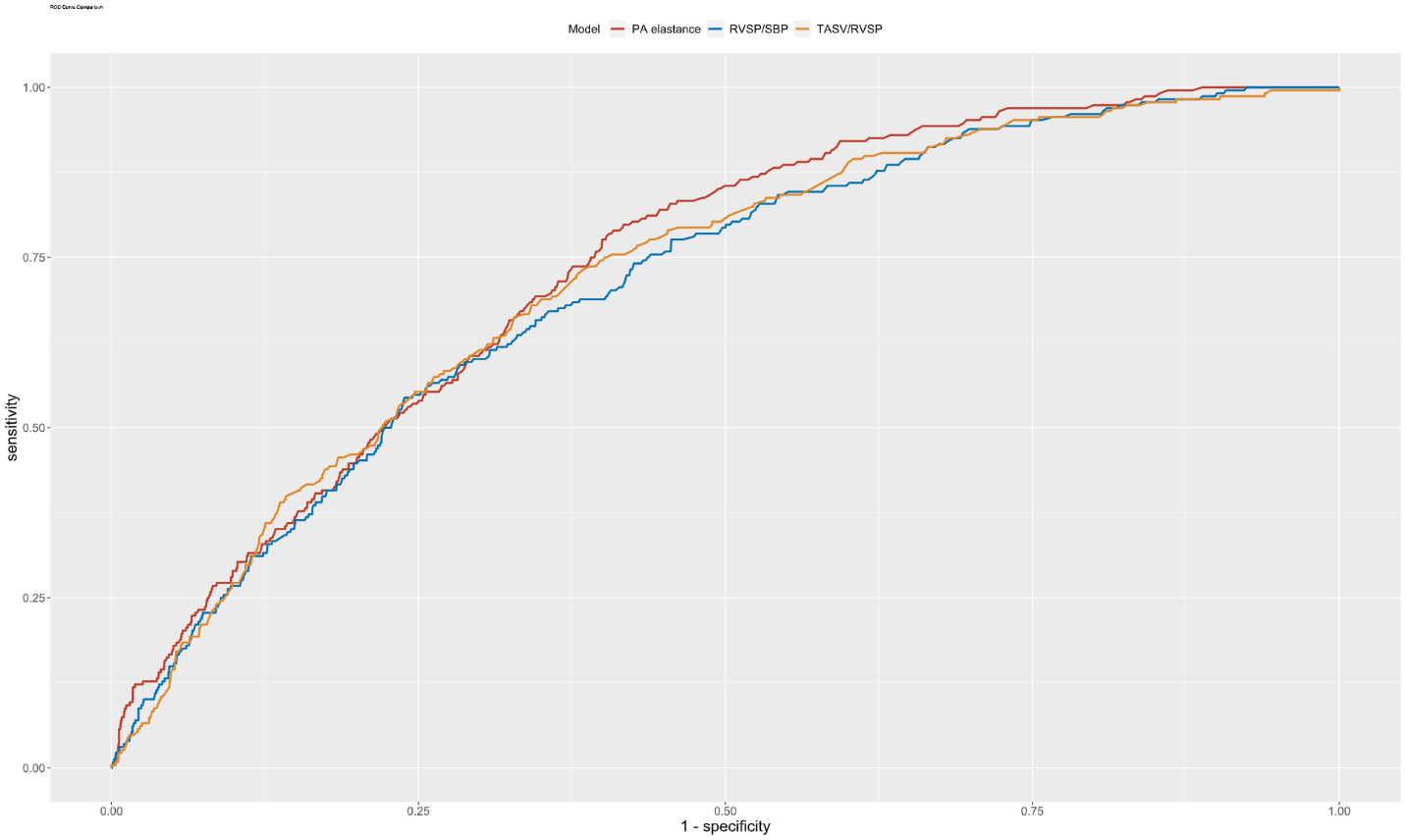


**Supplemental Figure 4:** In-hospital mortality according to quartiles of RVSP and SV (a) or SBP (b).


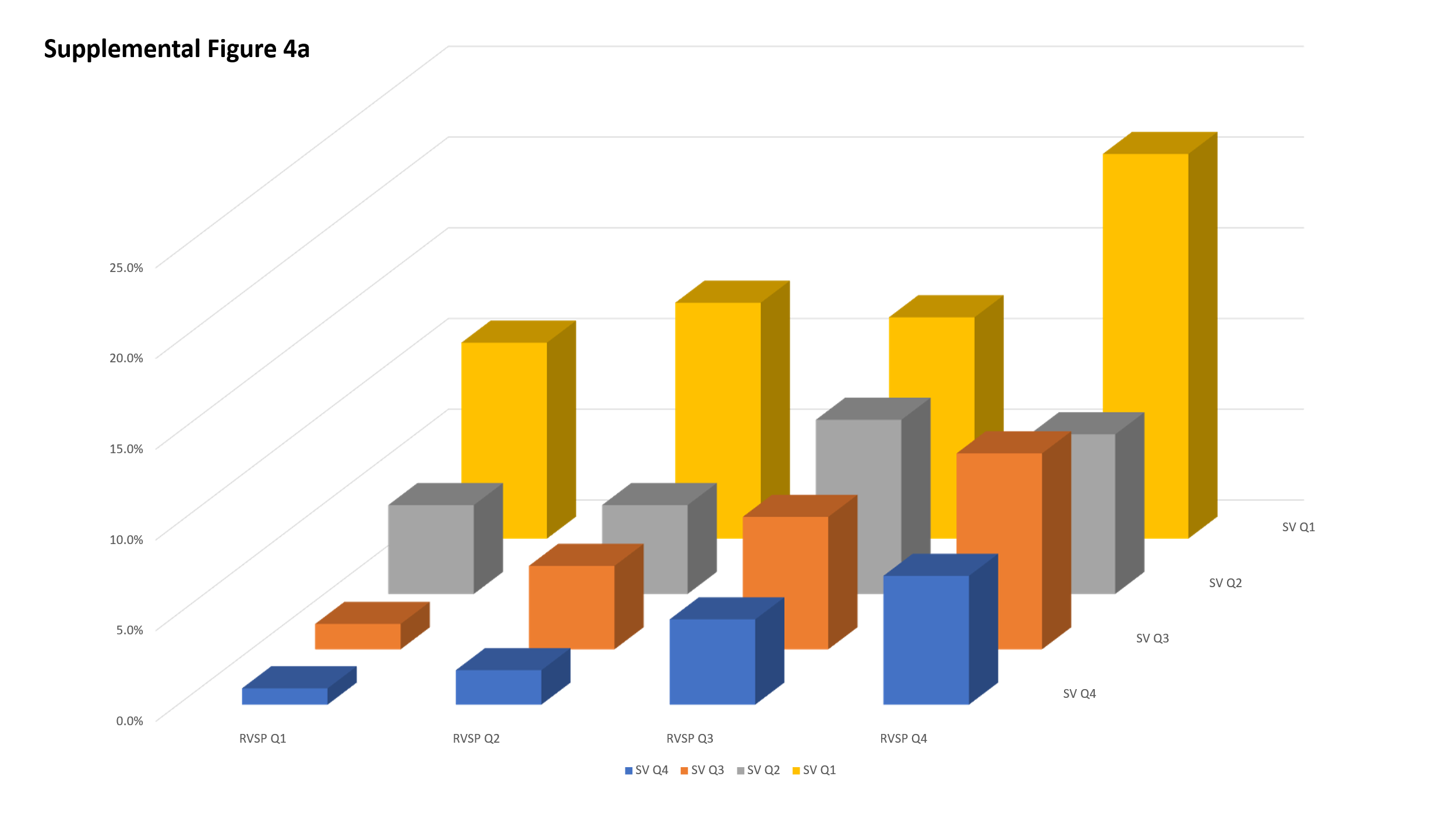


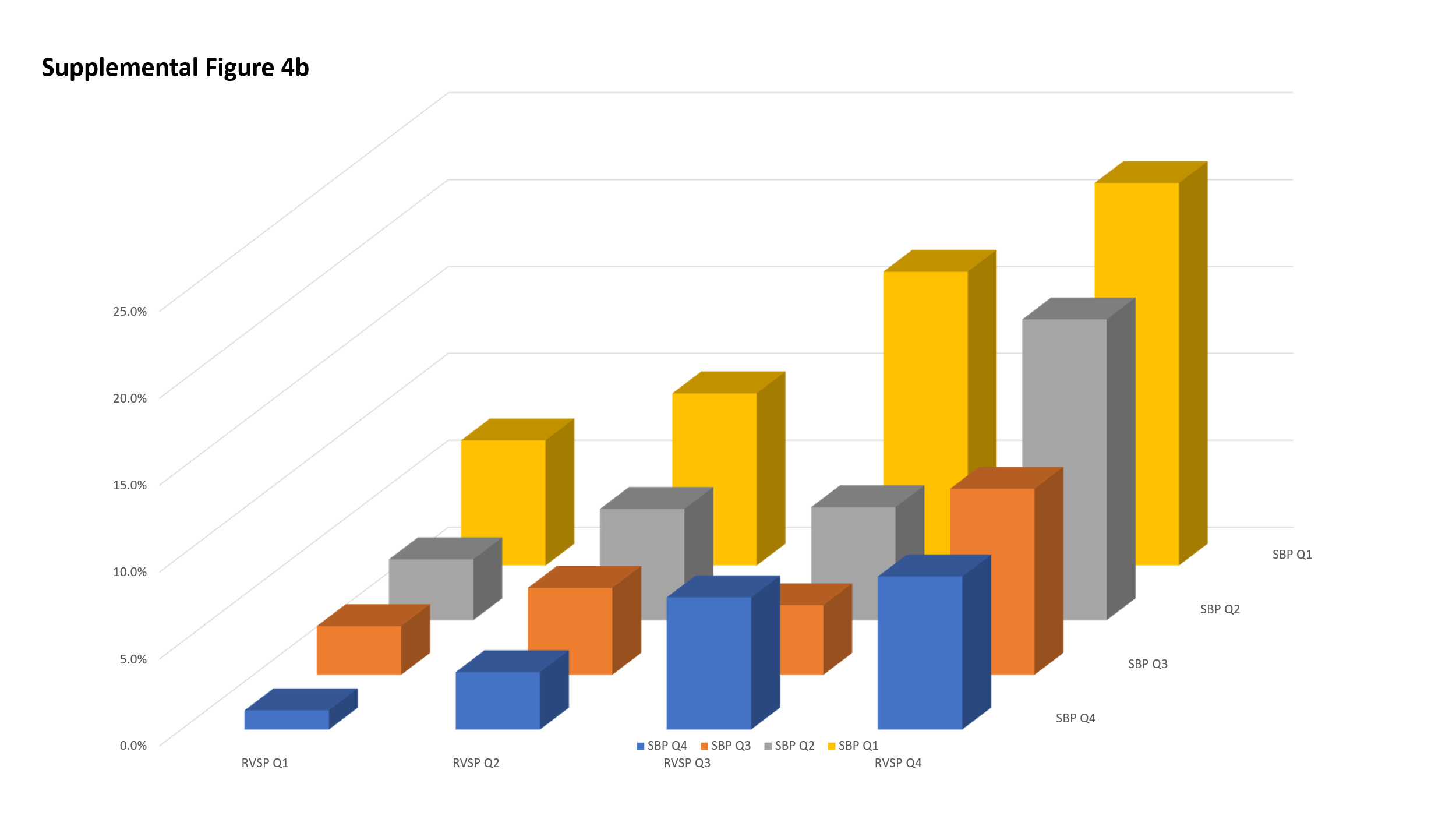


**Supplemental Figure 5:** In-hospital mortality according to left ventricular dysfunction stratified by E_PA_ quartiles (a) and RVSP/SBP quartiles (b).


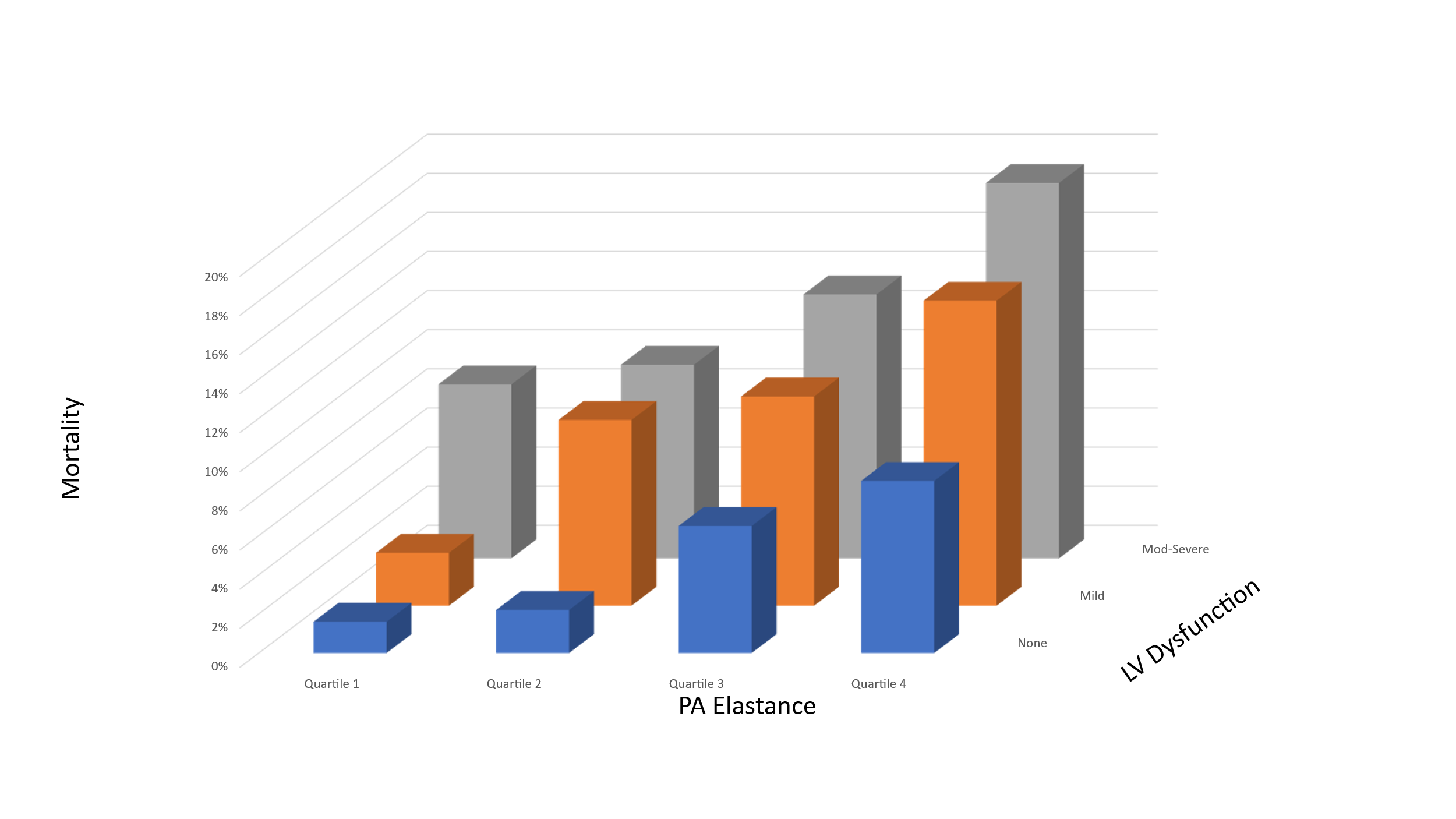


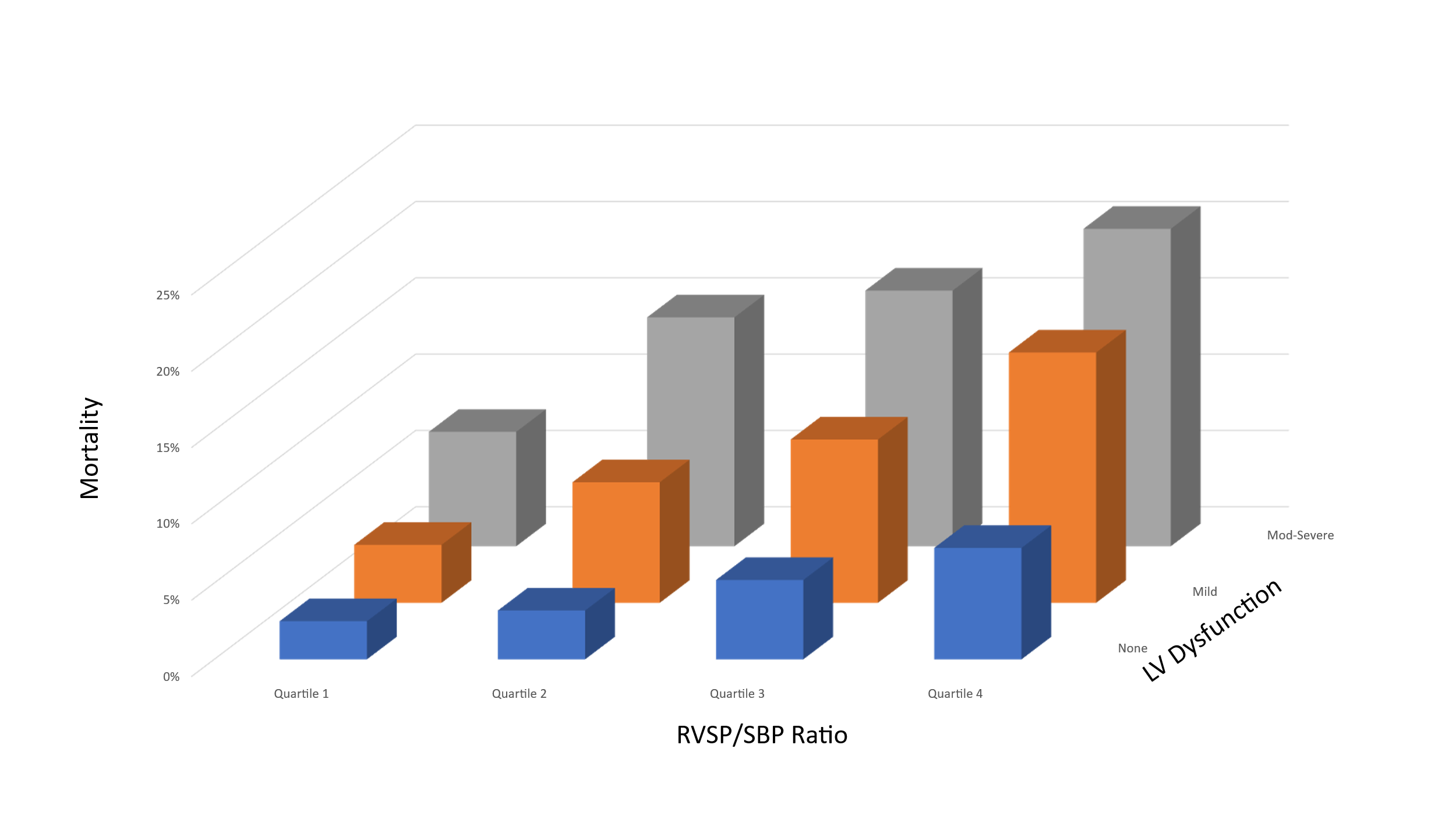

Supplement: Supplemental material [file mmc1.docx]
